# Characterization of a Spontaneous Retinal Neovascular Mouse Model

**DOI:** 10.1371/journal.pone.0106507

**Published:** 2014-09-04

**Authors:** Eiichi Hasegawa, Harry Sweigard, Deeba Husain, Ana M. Olivares, Bo Chang, Kaylee E. Smith, Amy E. Birsner, Robert J. D’Amato, Norman A. Michaud, Yinan Han, Demetrios G. Vavvas, Joan W. Miller, Neena B. Haider, Kip M. Connor

**Affiliations:** 1 Angiogenesis Laboratory, Department of Ophthalmology, Massachusetts Eye & Ear Infirmary, Boston, Massachusetts, United States of America; 2 Department of Ophthalmology, Harvard Medical School, Boston, Massachusetts, United States of America; 3 Schepens Eye Research Institute, Massachusetts Eye & Ear Infirmary, Boston, Massachusetts, United States of America; 4 The Jackson Laboratory, Bar Harbor, Maine, United States of America; 5 Vascular Biology Program, Department of Surgery, Boston Children’s Hospital, Harvard Medical School, Boston, Massachusetts, United States of America; Indiana University College of Medicine, United States of America

## Abstract

**Background:**

Vision loss due to vascular disease of the retina is a leading cause of blindness in the world. Retinal angiomatous proliferation (RAP) is a subgroup of neovascular age-related macular degeneration (AMD), whereby abnormal blood vessels develop in the retina leading to debilitating vision loss and eventual blindness. The novel mouse strain, neoretinal vascularization 2 (NRV2), shows spontaneous fundus changes associated with abnormal neovascularization. The purpose of this study is to characterize the induction of pathologic angiogenesis in this mouse model.

**Methods:**

The NRV2 mice were examined from postnatal day 12 (p12) to 3 months. The phenotypic changes within the retina were evaluated by fundus photography, fluorescein angiography, optical coherence tomography, and immunohistochemical and electron microscopic analysis. The pathological neovascularization was imaged by confocal microscopy and reconstructed using three-dimensional image analysis software.

**Results:**

We found that NRV2 mice develop multifocal retinal depigmentation in the posterior fundus. Depigmented lesions developed vascular leakage observed by fluorescein angiography. The spontaneous angiogenesis arose from the retinal vascular plexus at postnatal day (p)15 and extended toward retinal pigment epithelium (RPE). By three months of age, histological analysis revealed encapsulation of the neovascular lesion by the RPE in the photoreceptor cell layer and subretinal space.

**Conclusions:**

The NRV2 mouse strain develops early neovascular lesions within the retina, which grow downward towards the RPE beginning at p15. This retinal neovascularization model mimics early stages of human retinal angiomatous proliferation (RAP) and will likely be a useful in elucidating targeted therapeutics for patients with ocular neovascular disease.

## Introduction

Vision loss due to vascular disease of the retina is a leading cause of blindness in the world and affects all age groups. Neovascularization is a hallmark of complex, polygenic diseases, such as retinopathy of prematurity, proliferative diabetic retinopathy, and age-related macular degeneration (AMD). AMD, affects 1 in 3 individuals over age 65, occurs in two forms: exudative (“wet”) and nonexudative (“dry”). Advanced exudative AMD is defined by the formation of abnormal blood vessels that grow from the choroidal vasculature, through breaks in Bruch’s membrane, into the outer retina [1]. Less commonly, the abnormal vessels originate from retinal vasculature and can form a retinal–choroidal anastomosis (RCA) with the choroidal vascular bed [1]–[4]. These blood vessels are immature and leaky, resulting in subretinal and intraretinal edema and hemorrhage, which leads to vision loss [1], [5]. Neovascular AMD accounts for 10–15% of AMD cases, and if left untreated, rapidly leads to substantial vision loss [1], [6]. In recent years, a subgroup of neovascular AMD coined as retinal angiomatous proliferation (RAP) has been described where the disease starts as neovessels originating from the retinal vascular bed and grow towards the outer retina, ultimately forming choroidal neovascularization [7]–[9]. RAP [9], has been classified into three stages: stage I, when intraretinal neovascularization (IRN) arises from the deep capillary vascular plexus within the retina; stage II, is determined by growth of these abnormal retinal vessels into the subretinal space to form subretinal neovascularization (SRN); and Stage III occurs when choroidal neovascularization is observed, retinal–choroidal anastomosis (RCA) is a persistent feature of this stage. While it is well known that RAP follows a different natural course and response to treatment from typical neovascular AMD [10], there are few established models that allow us to fully understand the disease pathophysiology or to test new treatments for RAP [11], [12].

There are several animal models of retinal neovascularization [13]–[17], however, the precise molecular mechanism of RAP remains to be elucidated. The neoretinal vascularization (NRV) 2 mouse line, also called JR5558 mice, was discovered through The Jackson Laboratory Eye Mutant Screening program, which has identified a large number of new mouse models that exhibit unique eye phenotypes [18]. In this study, we characterize a novel mutant mouse model, NRV2, which appear to have a recessive mode of inheritance characterized by multiple areas of retinal depigmentation and vascular leakage in the posterior fundus. In this study, we focused on the characterization of the early ocular phenotype and etiology of angiogenesis in NRV2 mice. We discovered that depigmentation and neovascularization occur spontaneously and are concurrent with the presence of the abnormal blood vessels in the photoreceptor cell layer of the retina. Furthermore, NRV2 neovascularization originates from the retinal vascular plexus and grows outwards to the subretinal space, forming neovascularization structures at the RPE Bruch’s membrane interface and mimicking the early clinical presentation of RAP in humans. This genetic model of retinal neovascularization will provide a significant tool that will aid in our understanding of the molecular causes of retinal neovascular disease as well as in developing preclinical therapeutics.

## Materials and Methods

### Animals

JR5558 mice were obtained from The Jackson Laboratory (Bar Harbor, ME) and named neoretinal vascularization 2 (NRV2) mice. As far as we have observed, there is close to 100% penetrance of the ocular phenotype in this strain and we have not found any other gross abnormalities in other organ systems to date. All animal procedures adhered to the Association for Research in Vision and Ophthalmology Statement for the Use of Animals in Ophthalmic and Vision Research. The Animal Care and Use Committee of Massachusetts Eye and Ear Infirmary approved the protocol for experiments outlined herein. The n’s expressed are reflective of an individual mouse, not an individual eye.

### Fundus photography

Fundus images were taken using the Micron III Retinal Imaging Microscope (Phoenix Research Laboratories, Pleasanton, CA). Mice were anesthetized with Avertin (Sigma, St. Louis, MO) and placed on the 37°C heating pad to keep body temperature. The pupils were dilated with 5% phenylephrine and 0.5% tropicamide. Images were taken by attaching cornea covered with 2.5% Goniovisc (HUB Pharmaceuticals, Rancho Cucamonga, CA) to the Micron camera lens using the StreamPix software (Phoenix Research Laboratories, Pleasanton, CA).

### Fluorescein angiography

Fluorescein angiography (FA) was performed using a Micron III Retinal Imaging system directly following imaging of the fundus. Fluorescein images were captured at 3–5 minutes after i.p. injection of 0.1 ml of 2% fluorescein sodium (Akorn, Lake Forest, IL) and used to quantify the number of the vascular leakage areas. The images were captured at 1, 3–5 and 10 minutes after injection to define the time course of vascular leakage over time.

### Spectral Domain Optical Coherence Tomography (SD-OCT)

OCT images were taken using the Micron III Retinal Imaging Microscope. OCT images were taken by line scan under the guide of bright or Fluorescein field. The line scan is made up of a series of 1024 single point A-scans.

### Histology

Eyes were enucleated and fixed with Methanol/Acetic acid (3∶1) overnight at 4°C. Tissues were paraffin embedded. Serial 5 µm thick sections were cut and stained with hematoxylin and eosin (H&E), examined under bright field light microscopy.

### Immunohistochemical Staining and 3D reconstruction of retinal vascular structure

Eyes were enucleated and fixed in 4% paraformaldehyde for 1 hour at room temperature. Retinas were isolated and blocked in PBS with 0.5% Triton X-100 and 5% goat serum. Retinas were rinsed in PBS, permeabilized overnight at 4°C with 0.5% Triton X-100 in PBS, and stained overnight with FITC-conjugated G. simplicifolia isolectin B4 (Alexa Fluor 488-I21411, 1∶100 dilution; Life Technologies, Carlsbad, CA) in 1 mM CaCl_2_ in PBS. After 2 hours of washes, retinas were whole-mounted onto microscope slides (Superfrost/Plus; Fisher Scientific, Pittsburgh, PA) with the photoreceptor side down and embedded in reagent (PermaFluor; Thermo Scientific, Fremont, CA). Retinal wholemounts were visualized with a confocal microscope (SP5; Leica, Wetzlar, Germany). Retinal vascular structures were reconstructed with Amira 5.2 software (Visualization Sciences Group, Burlington, MA) from the pictures taken by the confocal microscope.

### Electron microscopy

Eyes were enucleated and cleaned of all extraneous tissue then rinsed with saline. Eyes were immediately placed into fixative consisting of 2.5% gluteraldehyde and 2% formaldehyde in 0.1 M cacodylate buffer with 0.08 M CaCl_2_ at 4°C. After a short 10 to 15 minute fixation, eyes were bisected at the limbus and the anterior segment was separated from the posterior segment and the parts to be examined were placed back in the fixative. Within 24 hours of enucleation, the tissues were washed in 0.1 M cacodylate buffer and stored at 4°C. The tissues were post-fixed for 1.5 hours in 2% aqueous OsO_4_. The tissues were dehydrated in graded ethanols, transitioned in propylene oxide, infiltrated with propylene oxide and epon mixtures (tEpon, Tousimis) embedded in epon and cured for 24–48 hours at 60°C. One-micron sections were cut on a Leica Ultracut UCT and stained with 1% toluidine blue in 1% borate buffer. Areas selected from the 1 micron thick sections were further sectioned at 70–90 nm, stained with saturated Uranyl Acetate and Sato’s lead stain and examined with a Philips CM-10 electron microscope.

### ELISAs for VEGF and sFLT-1

The retina tissue was homogenized with a lysis buffer (Roche Diagnostics) containing protease inhibitors (Roche Diagnostics), and the homogenates were centrifuged at 16,200×*g* for 10 min at 4°C. The amounts of VEGF and sFLT-1 protein were assayed using ELISA systems (R&D Systems).

### Statistical analysis

Data are presented as means ± SEM and were analyzed with Student t test for comparisons between two groups. P value of <0.05 was considered statistically significant.

## Results

### Depigmentation and vascular leakage observed in retinas of NRV2 mice

**Figure 1:**
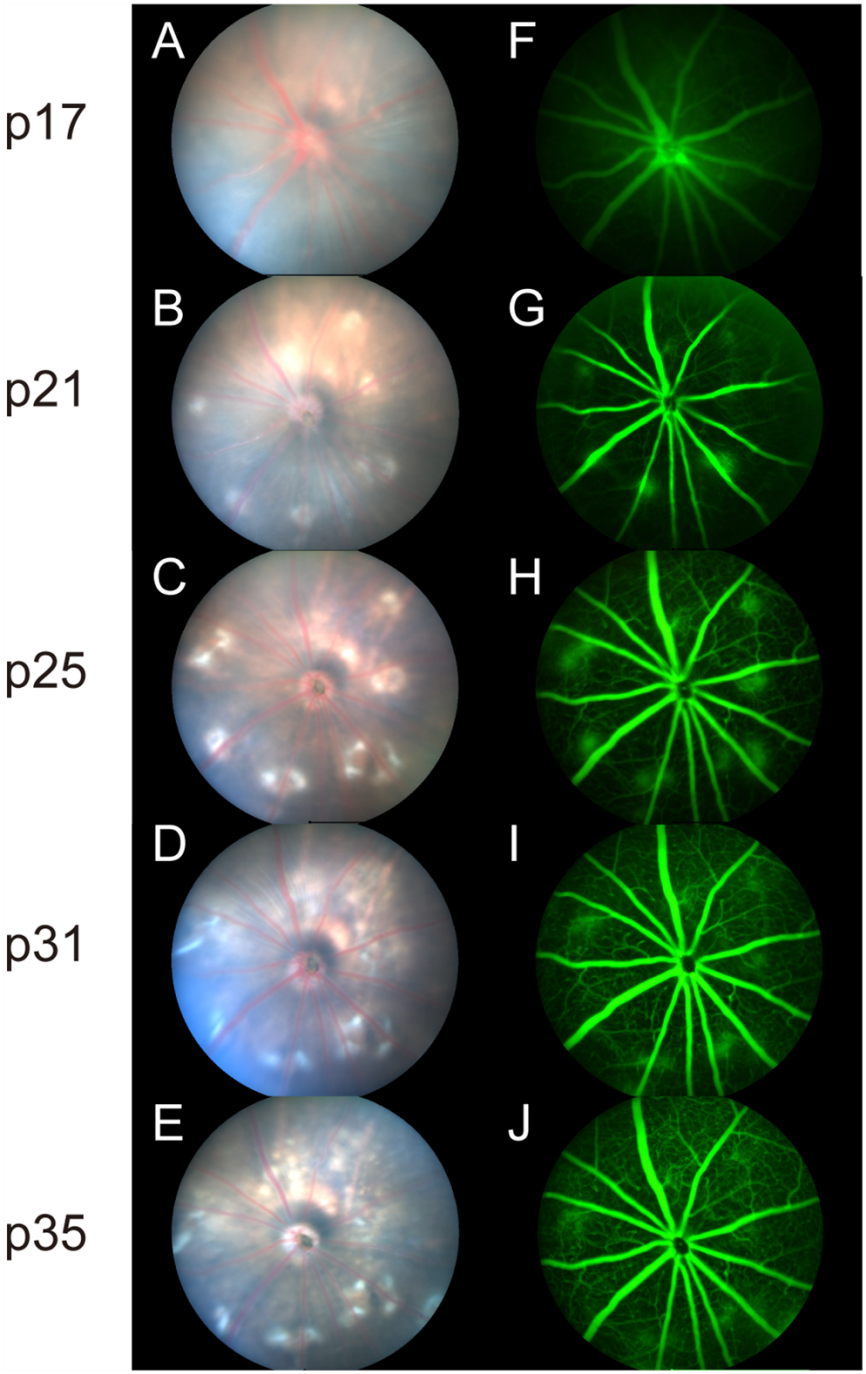
Time course representing the morphological changes found in the fundus of NRV2 mice. Fundus photographs and fluorescein angiography of NRV2 mice from p17 to p35. (*A*–*E*) Fundus images show the emergence of depigmented regions at p17 that increase in size and number through p35. (*F*–*J*) Fluorescein angiography indicates vascular leakage corresponding to the areas of depigmentation, peaking at p25 and subsiding by p35. n = 10, Representative images are shown. p = postnatal day.

**Figure 2:**
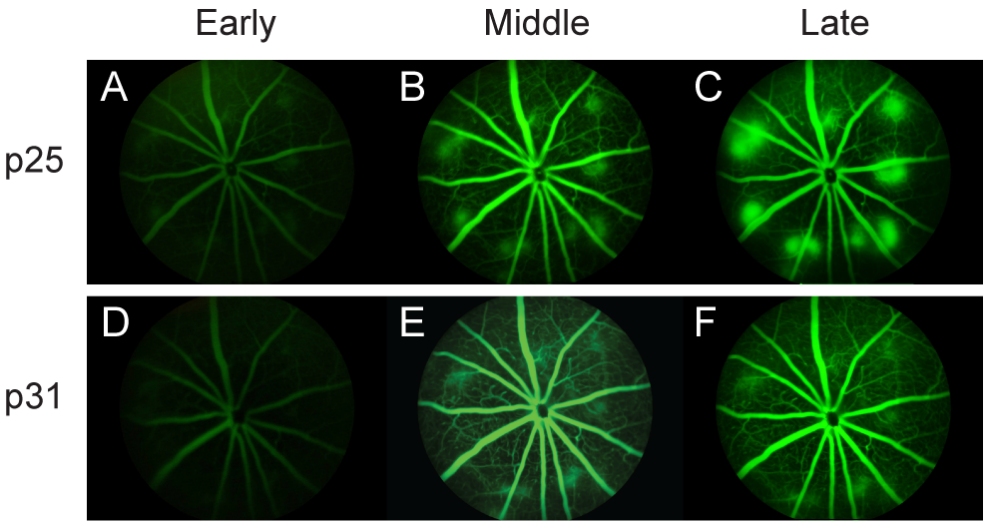
Vascular leakage in retinal lesions of NRV2 mice during postnatal development. Fluorescein angiography images taken of NRV2 mice at the early through late phases after fluorescein injection for p25 and p31. (*A*–*C*) At p25 NRV2 mice have increased vascular leakage through the late phase of fluorescein angiography, compare B and C. (*D*–*F*) By p31, there is reduced vascular leakage relative to p25, that does not increase from the middle to late phase of fluorescein angiography, compare E and F. Early = 1 minute after fluorescein injection, middle = 3–5 minutes after fluorescein injection, late = 10 minutes after fluorescein injection. n = 10, Representative images are shown. p = postnatal day.

**Figure 3:**
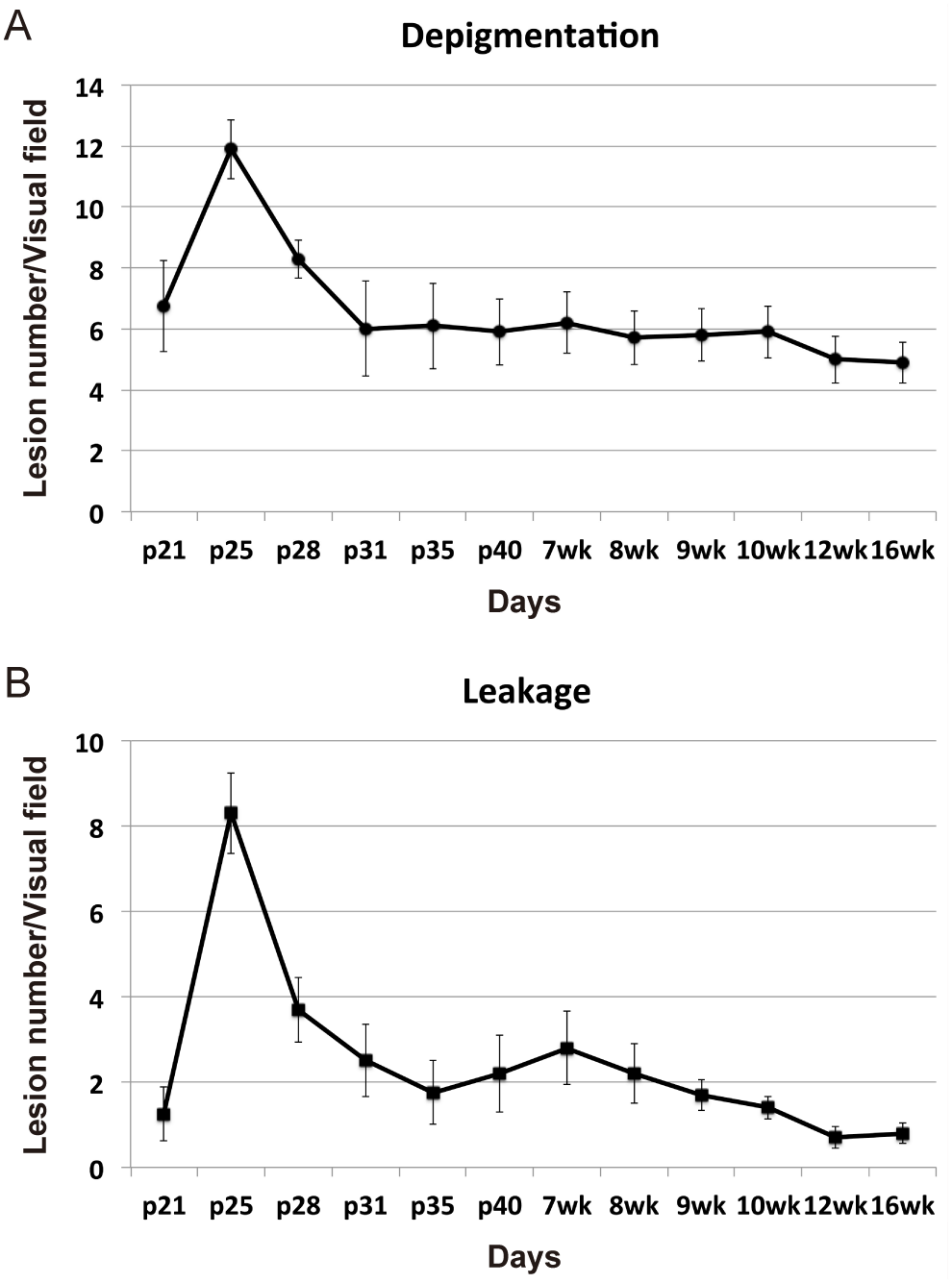
The peak time for depigmentation and vascular leakage in NRV2 mice is at p25. Developmental time course of depigmentation and vascular leakage assessed by funduscopy and fluorescein angiography in NRV2 mice. (*A*) Quantitation of the number of depigmented regions observed by fundus imaging. The peak number of depigmented regions occurs at p25, followed by a relative stabilization. (*B*) Quantitation of the number of fluorescein angiography leakage, measured by the number of leakage regions, also peaks at p25. Each plot on the graph is an average of 10 eyes, p = postnatal day.

A longitudinal study was conducted to determine the onset and progression of clinical manifestations of neovascular disease in NRV2 mice. Fundus exams were performed using a fundus imaging microscope at postnatal day p17 through postnatal day p35. Diffuse areas of depigmentation were observed in the retina beginning at p17 (Fig. 1A, Fig. S1A, B). Depigmented areas begin to become more focal in nature and increase in number at p21 (Fig. 1B). The lesions become well demarcated and larger in size at p25 (Fig. 1C). After p25, these areas become more diffuse in nature (Fig. 1D, E, Fig. S1E, F). Vascular leakage was detected by fluorescein angiography (FA). No leakage is observed at p17 (Fig. 1F, Fig. S1C, D). The vascular leakage pattern follows the appearance of retinal depigmentation becoming evident at p21 (Fig. 1G) and peaks at p25, corresponding with the height of focal depigmentation in these animals (Fig. 1H). The areas of depigmentation correlated with the development of vascular leakage, indicating a vascular component to this disease phenotype. As with the focal depigmentation, fluorescein leakage gradually decreased after p25 (Fig. 1I, J, Fig. S1G, H). Early, moderate and late phase fluorescein angiograms were performed to determine the degree of vascular leakage in NRV2 mice. At p25, early leakage was observed that became both brighter and larger in size as time progressed to the late phase of the angiogram, indicating active neovascularization (Fig. 2A–C). At p31, minimal leakage was observed from the onset of the angiogram, which persisted through the late phase without any further increase in leakage (Fig. 2D–F), indicating that vascular leakage develops fairly early in these mice at p25 and begins to resolve by p31. Longitudinal fundus analysis from p21 through 16 weeks revealed both the number of depigmentation areas (Fig. 3A) and FA leakage areas (Fig. 3B) peaked at p25. By day 31 the areas of depigmentation became more diffuse and enlarged, but the vascular leakage largely subsided (Fig. 3A, B).

### Spectral domain optical coherence tomography reveals dysmorphic outer and inner retinal layers

**Figure 4:**
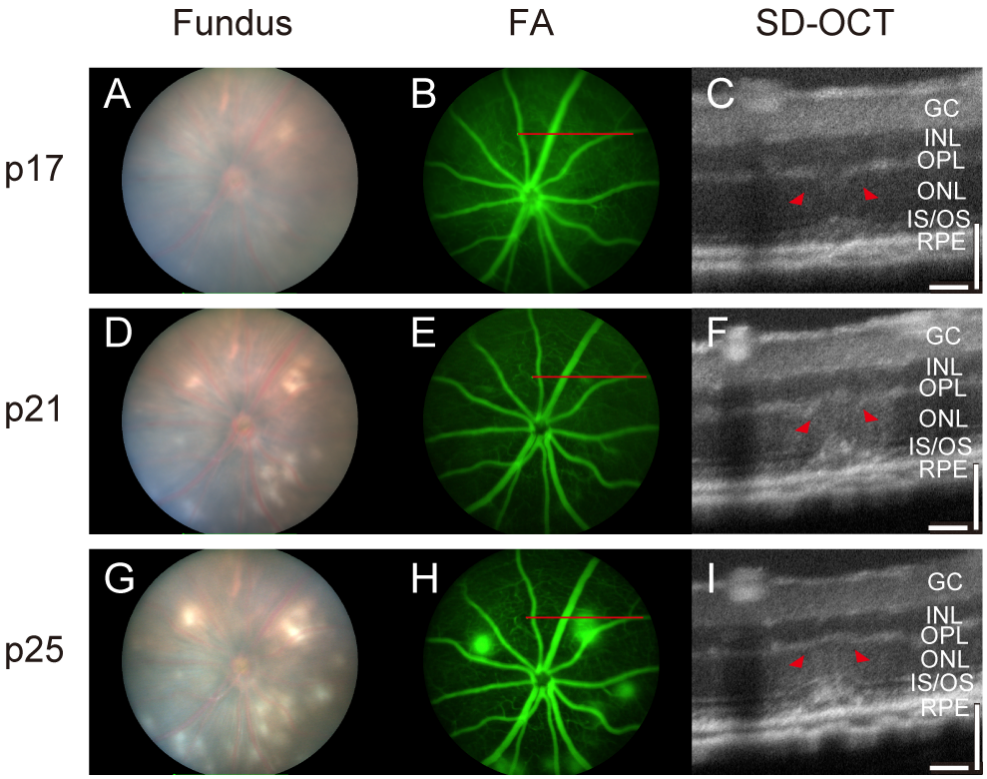
Vascular leakage corresponds to disturbances in the outer plexiform and outer nuclear layers of the retina. Images showing the representative time course of lesion development in a NRV2 mouse. (*A*–*C*) Fundus image taken at p17, showing areas that have slight depigmentation, at the 1 o’clock position of the image (*A*, left panel). In the same area, vascular leakage can be seen by fluorescein angiography (*B*, middle panel). Examination by SD-OCT at the region of vascular leakage (red bar in *B*) reveals a disturbance in the ONL and OPL of the retina (*C*, right panel). Arrowheads point to the retinal disturbance. (*D*–*F*) Fundus image taken at p21, of the same mouse, shows increased depigmentation at the 1 o’clock position of the image (*D*, left panel). In the same area vascular leakage can be seen by fluorescein angiography (*E*, middle panel). Examination by SD-OCT at the region of vascular leakage (red bar in *E*) reveals a disturbance in the ONL and OPL of the retina (*F*, right panel). Arrowheads point to the retinal disturbance. (*G*–*I*) Fundus image of the same mouse taken at p25, the height of leakage in NRV2 mice, shows depigmentation, at the 1 o’clock position of the image (*G*, left panel). In the same area, vascular leakage is pronounced compared to the earlier time points as seen by fluorescein angiography (*H*, middle panel). Examination by SD-OCT at the region of vascular leakage (red bar in *H*) reveals a disturbance in the ONL and OPL of the retina (*I*, right panel). Arrowheads point to the retinal disturbance. p = postnatal day, GC: ganglion cells; INL: inner nuclear layer; OPL: outer plexiform layer; ONL: outer nuclear layer; IS/OS: photoreceptor inner segment/outer segments. n = 10, Representative images are shown. Scale bars: 50 µm.

Spectral domain optical coherence tomography (SD-OCT) allows for a detailed, noninvasive evaluation of the retinal architecture *in vivo*, and has been found to accurately reflect retinal morphological changes that occur during retinal disease progression. We followed lesion development in the NRV2 mouse by SD-OCT at p17, p21, and p25. Depigmentation areas first appear at p17, however, no vascular leakage is evident on FA (Fig. 4A, B). In the SD-OCT, a disruption is apparent in the outer plexiform layer (OPL) and a highly reflective lesion is present in the inner segment/outer segment (IS/OS) (Fig. 4C). As the lesion develops from p21–p25, the depigmentation area becomes more prominent (Fig. 4D, G) and is associated with increased vascular leakage (Fig. 4E, H) The SD-OCT assessment reveals enlargement of the subretinal and inner segment/outer segment (IS/OS) zone along with a highly reflective area and persistent irregularity of the ONL and OPL (arrows, Fig. 4C, F, I).

### Retinal neovascular development in the NRV2 mouse originates in the inner retina and extends outwards to the RPE

**Figure 5:**
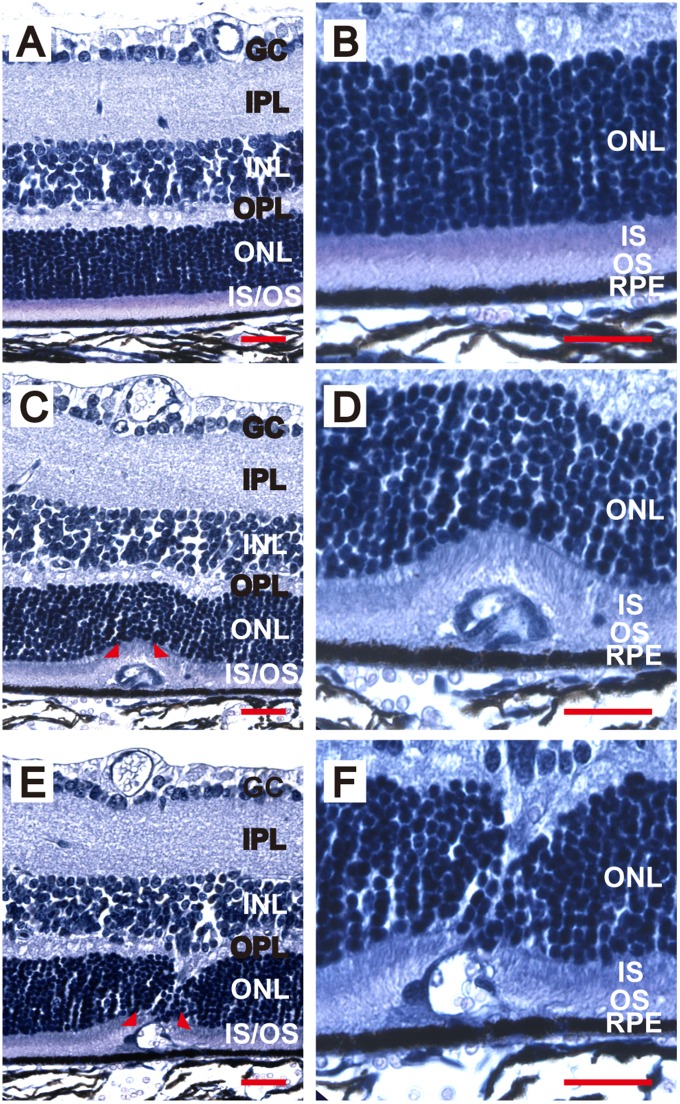
Neovessels extend from the INL to the RPE in NRV2 mice. Retinal cross-sections stained by H&E through a neovascular lesion. (*A*) Cross-section from a C57Bl6 mouse showing the normal architecture of the retinal layers. (*B*) Higher magnification of (*A*) focusing on the normal architecture between the ONL and RPE interface. (*C*) Cross section from an NRV2 mouse at p25, showing a disturbance in the outer retina. (*D*) Higher magnification of (*C*) to show that the lesion is at the interface of the ONL and RPE, in the outer segments of the photoreceptors. (*E*) Retinal cross-section from another NRV2 mouse at p25, capturing the track of a neovessel spanning from the INL to the RPE. (*F*) Higher magnification of (*E*) showing the vessel extending through the ONL. Arrowheads in (*C)* and (*E*) indicate the lesion areas that are magnified in (*D*) and (*F*). GC: ganglion cells; IPL: inner plexiform layer; INL: inner nuclear layer; OPL: outer plexiform layer; ONL: outer nuclear layer; IS/OS: photoreceptor inner segment/outer segments. n = 3, Representative images are shown. Scale bars: 25 µm.

**Figure 6:**
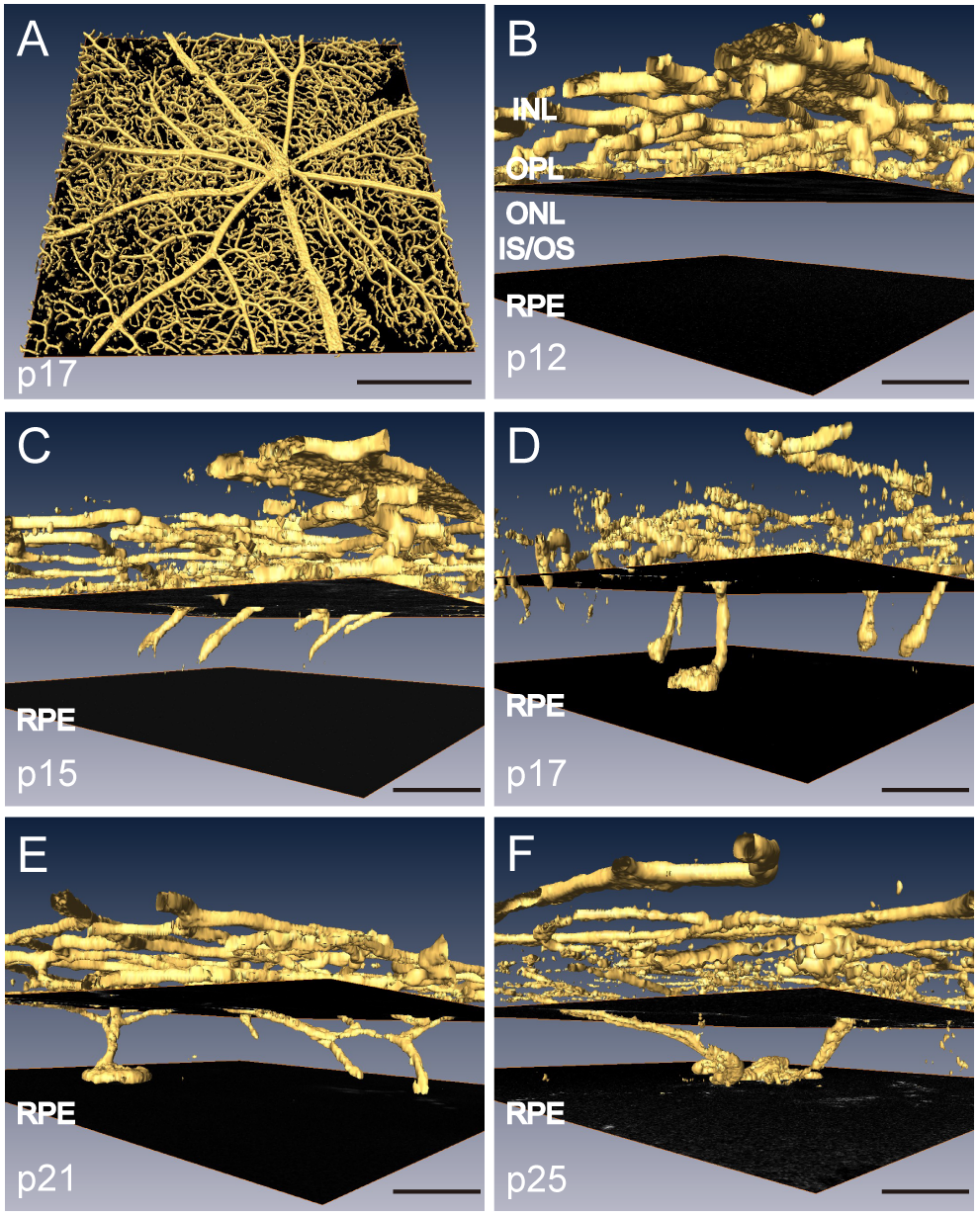
Neovessels originate from the INL and grow towards the RPE in NRV2 mice. Retinal cross-sections stained with isolectin B4 that have been reconstructed into 3D images (Amira software) using confocal Z stacks to show the development of retinal blood vessels in NRV2 mice. (*A*) Low magnification overview of the 3D reconstruction of a retinal flatmount stained with isolectin B4 for blood vessels. (*B*–*F*) High magnification images of retinal cross-sections from NRV2 mice stained with isolectin B4 that have been reconstructed into a 3D montage. (*B*) At p12, there are only vessels in the INL with no apparent irregular vessel development. (*C*) At p15, vessels are found extending from the INL towards the RPE cell layer. (*D*) At p17, the vessels have reached the RPE interface. (*E*) At p21, vessels have mostly interfaced with the RPE cell layer and have begun to expand across the surface of the RPE. (*F*) At p25, the vessels have fully interacted with the RPE and formed balloon like structures. p = postnatal day, INL: inner nuclear layer; OPL: outer plexiform layer; ONL: outer nuclear layer; IS/OS: photoreceptor inner segment/outer segments. The upper black square shows the border of the OPL and ONL. The lower black square shows the interface of the RPE layer. n = 3/timepoint, Representative images are shown. Scale bars: (*A*): 500 µm, (*B–F*): 50 µm.

Fundus characterization of the NRV2 eye strongly indicates the presence of neovascular structures within retina. Retinal histology was performed on NRV2 ocular tissue to determine the origin of the neovascularization. Normal retina (Fig. 5A, B and Fig. S2) was compared to retinal areas with lesions in the NRV2 mouse. Retinal cross-sections revealed the presence of lesions containing a lumenal structure near photoreceptor outer segments (Fig. 5C, D). The abnormal vascular structure appears to originate from the deep layer of the retinal vascular plexus, at the outer edge of the inner nuclear layer (INL). Neovessels descend into the outer nuclear layer (ONL), which is typically an avascular area, and then reach the RPE and spread, initially, above the RPE (Fig. 5E, F). The RPE and choriocapillaris appear normal in these sections. These findings are supported by our SD-OCT images, which show abnormal structures originating from the OPL and extending into the subretinal space (Fig. 4C, D). However, it still remained unclear if these vessels originated from the retinal vasculature or if they were of choroidal origin, which subsequently anastamosed to the outer retinal plexus. We assessed the formation of the subretinal neovascular lesions in retinal flatmounts stained with isolectin to gain a more complete understanding of the origin of these vessels. A confocal z-stack was taken through the retina and the vasculature was reconstructed into three-dimensional models using Amira software. We assessed eyes at p12, p15, p17, p21 and p25 in order to capture the development of these lesions. At p12, normal reticulated retinal vasculature was present in the INL and no abnormal vessels were observed in the ONL (Fig. 6B). In contrast, beginning at p15, neovessels begin to grow and extend into the ONL from the INL (Fig. 6C, movie S1). These vessels continued to extend downward until they reach the RPE, where they balloon above the RPE from p17 through p25 (Fig. 6C–E, movie S2, S3).

### Transmission electron microscopy (TEM) of NRV2 reveals lesion architecture and development

**Figure 7:**
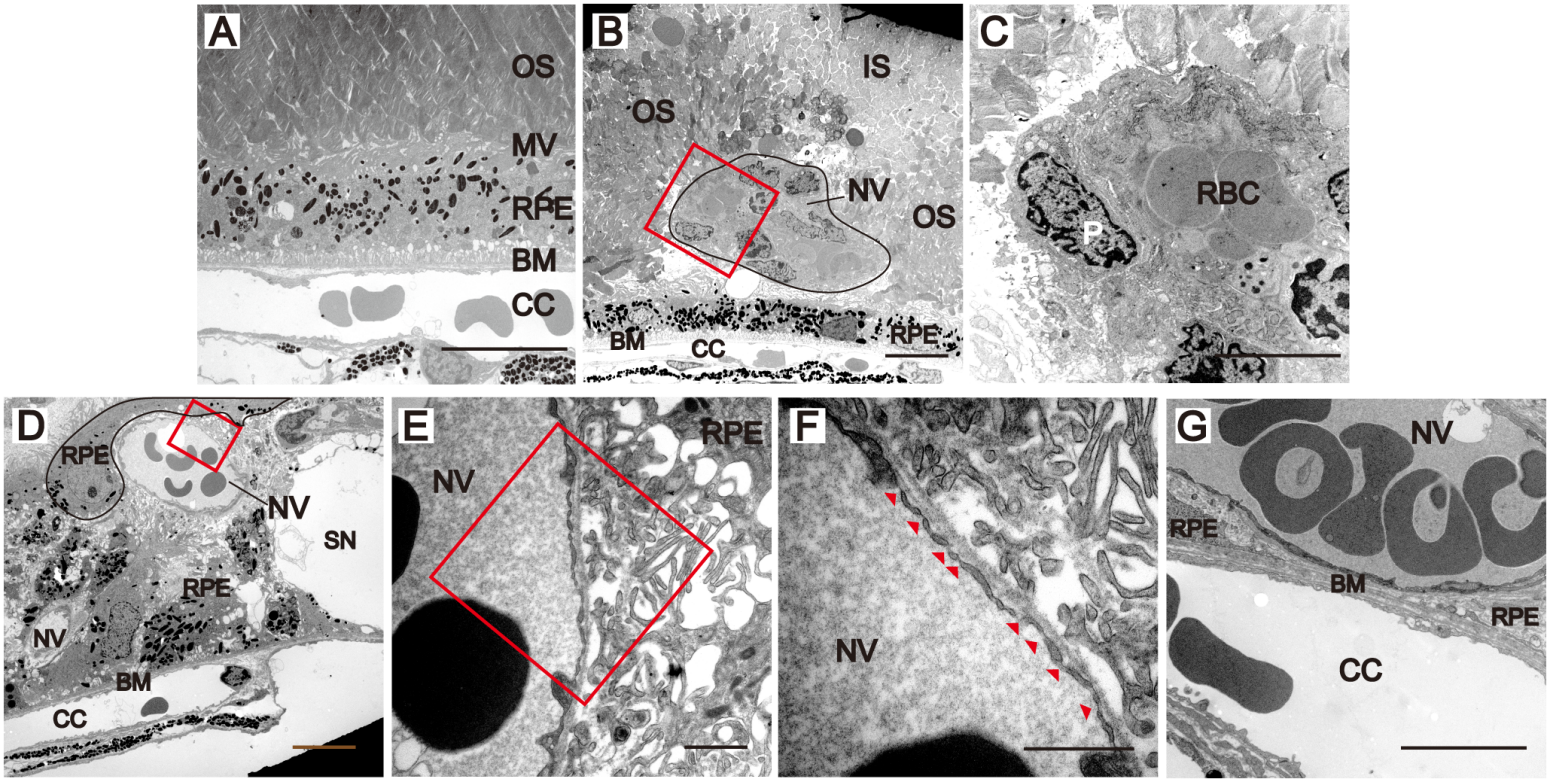
Neovessels that interface with the RPE form characteristic morphological features of more mature vessels. Electron micrographs taken of NRV2 mice illustrate commonly observed morphological findings of neovessels when they come in contact with the RPE/Bruch’s membrane. (*A*) Electron micrograph of a C57BL/6 mouse at 3 months of age shows the normal architecture of the outer retina. (*B*) Neovessels (outlined in black) from an NRV2 mouse at p21, surrounded by disorganized outer photoreceptor segments. (*C*) High magnification image (red box in *B*) that shows the presence of pericytes in the neovessels. (*D*) A 3 months old, NRV2 mouse with an RPE cell (outlined in black) surrounding the neovessel. (*E*) High magnification (red box in *D*) that shows a contact point between the neovessel and RPE cell. (*F*) Higher magnification (red box in *E*) of the contact point reveals fenestrations (arrowheads). (*G*) A 3 months old, NRV2 mouse where the neovessel has come in direct contact with the Bruch’s membrane. IS: inner segment; OS: outer segment; MV: microvilli; BM: Bruch’s membrane; CC: choriocapillaris; NV: new vessels; P: pericyte; n = 3/timepoint, Representative images are shown. Scale bars: (*A*–*D, G*): 5 µm, (*E, F*): 1 µm.

Morphological features at the RPE-Bruch’s interface were assessed using electron microscopy to further delineate ultrastructural characteristics of NRV2 neovascular lesions. The outer segments, RPE, Bruch’s membrane, and choriocapillaris are well organized in C57BL/6 mice (Fig. 7A). In contrast, new vessels are evident at the photoreceptor outer segment-RPE interface at p21 in NRV2 mice (Fig. 7B). Here the outer segments appear disorganized (damaged and or missing) and are lacking their typical morphologic structure (Fig. 7B). Two new vessels are observed among the outer segments above the RPE and choriocapillaris, which appear normal at p21 (Fig. 7B). Higher magnification of the lesion identifies pericytes that are clearly evident around the new vessels in these lesions (Fig. 7C). These new vessel structures are also observed in 3 months old NRV2 mice. However, by 3 months of age, the RPE layer is disrupted and proliferating RPE cells have enveloped the neovascular lesion (Fig. 7D).

Interestingly, there are infoldings of the RPE at the surface of the cell in contact with the new vessels (Fig. 7E). The new vessels in turn show fenestrations along this contact area, mimicking the usual RPE- choriocapillaris interaction (Fig. 7F). Some of these new vessels are located directly over the Bruch’s membrane without an intervening RPE layer (Fig. 7G). Bruch’s membrane appeared intact and the choriocapillaris seemed normal at all time points as seen by Fig. 1C, by light microscopy and TEM using step sections.

It is well known that vascular endothelial growth factor (VEGF) is a potent inducer of intraocular neovascularization [19]. Several clinical studies have demonstrated that Anti-VEGF monotherapy has a certain inhibitory effect to RAP progression [20]–[22]. On the other hand, soluble FLT-1 (also known as soluble VEGF receptor-1) is a natural inhibitor of VEGF, which is produced in the photoreceptor layer [23]. It binds VEGF and regulates VEGF-mediated angiogenesis [24]–[26]. Both suppression of sFLT-1 by neutralizing antibodies and conditional knockdown of sFLT-1 in photoreceptor cells increased VEGF levels and caused vessels to enter the normally avascular photoreceptor layer [23]. We assessed the levels of VEGF and sFLT-1 in the retina of NRV2 mice during disease progression. VEGF levels of NRV2 mice were significantly higher than those of C57BL/6 control mice (Fig. S3A). However, there was no significant difference between the sFLT-1 levels in the retina of NRV2 mice and those of C57BL/6 mice (Fig. S3B), suggesting that overexpression of VEGF in the retina is involved in the cause of retinal angiogenesis in NRV2 mice.

## Discussion

In the present study, we characterized a novel mouse model of ocular neovascular disease, NRV2, by fundus, histological, and ultrastructural analysis. As shown in Figure 1–3, NRV2 mice spontaneously develop retinal depigmentation and vascular leakage in the fundus during early postnatal days. The NRV2 phenotype is bilateral with no bias for gender observed. Additionally, gross observation revealed no differences in other organs. Histological analysis revealed abnormal vessel structures present from the outer plexiform layer to the retinal pigment epithelium (Fig. 5C–F). Moreover, three-dimensional reconstruction of the retinal vasculature at p15 clearly demonstrates that these neovessels originate from the deep retinal vascular plexus in the outer plexiform layer, which grow through the outer retina towards the subretinal space between p17–p25 (Fig. 6). SD-OCT examination captured balloon-like neovessel structures in the photoreceptor cell layer (Fig. 4C, F, I). We could not identify any vascular structures of choroidal origin and three-dimensional reconstruction images of vasculature did not show vessels growing up from the choroidal vascular bed into the retina’s ONL layer. Our findings indicate that the retinal origin of angiogenesis in NRV2 mice is consistent with stage 1 and stage 2 of human RAP as defined by Yannuzzi et al [9]. Contrary to our findings, Nagai N et al. concluded that the neovessels in this mouse model originate from choriocapillaris [27]. They identify vascular structures in the subretinal space by immunohistochemical and histological analysis. Our ultrastrucutural analysis revealed that NRV2 neovessels proliferate into the subretinal space, located between the RPE and photoreceptor cell layer (Fig. 7B). As the neovascular lesions age, the neovessels become encapsulated by RPE (Fig. 7D) and likely blocks the fluorescein leakage initially seen from p21 to p25 (Fig. 1). Moreover, it was also observed that the neovessels can descend to the border of Bruch’s membrane at three months with no RPE cells or processes between the vessels and Bruch’s membrane (Fig. 7G). However, no breaks in Bruch’s membrane were ever identified in serial light or electron microscopic sections and the choriocapillaris morphology was normal. These findings further suggest that neovessels in NRV2 mice are of retinal origin and the growth of the neovessels into the subretinal space corresponds to the stage 2 of human RAP. Müller cells play an important role for retinal homeostasis [28]. Recently, it has been shown that ablation of Müller cells results in intraretinal neovascularization and vascular leakage [29]. However, in NRV2 mice, GFAP positive glial cells accumulated close to the neovascular area in the retina [27]. They might have pro-angiogenic effects in this mouse strain because Müller cells are also known to be a source of VEGF and TNF in hypoxic or inflammatory conditions [30]. The role of Müller cells in NRV2 mice remains to be elucidated.

Interestingly, the NRV2 neovessels were found to have fenestrations similar to the choriocapillaris (Fig. 7E, F). Previous reports show that RPE cells play an essential role in the development and maintenance of fenestrated choroidal vasculature [31]. RPE cells are also reported to have the ability to induce fenestrations in endothelial cells of retinal vessels by encapsulating them [32]. Given these prior findings, we propose that these retinal neovessels originate from the deep retinal vascular plexus and extend downward through the retina until they reach the RPE where they proliferate until RPE cells encapsulate the lesions, isolating them from the retinal microenvironment.

Yannuzzi et al. proposed three-stages of retinal angiomatous proliferation in AMD [9]. They considered the origin of the neovascularization in RAP to be derived from the inner retina. In the early stage of RAP, angiomatous proliferation arises spontaneously from intraretinal capillaries. During the second stage, the intraretinal neovessels extends outwards into the subretinal space. Finally, choroidal neovascularization occurs and forms retinal-choroidal anastomoses. In contrast, Gass et al. [33] suggested that focal atrophy of the RPE and type 1 occult choroidal neovascularization under the RPE are the initial lesions of RAP. These lesions permit the retinal neovasculature to infiltrate into the outer retina and form chorioretinal anastomoses with the original type 1 occult choroidal vessel. Using immunohistochemistry, Shimada et al. demonstrated the existence of intraretinal neovascularization, but not choroidal neovascularization, in excised RAP specimens from patients with stage 2 RAP [34]. They also showed that both choroidal neovascularization and chorioretinal anastomosis existed in the stage 3 RAP specimens. Their histopahological findings supported Yannuzzi’s original hypothesis, in which RAP originates from the inner retina [9].

Several mouse models have been reported to show RCA, which is seen in stage 3 of RAP [35]–[37]. Very low-density lipoprotein receptor (VLDLR) knock out mice begin to show abnormal retinal blood vessels originating from the outer plexiform layer and grow into the subretinal space around p14 [35], which is similar to the process of NRV2 mice. In the VLDLR knock out mouse model, RCA occurs approximately at 10 months of age and RPE cells surround the vessels [36]. Additionally, photoreceptor degeneration and the loss of the ONL were also observed [36]. In contrast, the neovascularization in mice lacking CYP27A1, which is the principal cholesterol hydroxylase in the retina, occurred in both retina and choroid [37]. This angiogenesis process was detected around 1.5 months. The major abnormalities included dilated blood vessels in the OPL and thickened fibrovascular structures between the RPE and Bruch’s membrane. The RCA formation was observed at 11 months, which is later than VLDLR knock out mice. This angiogenesis process might be more representative of Gass’s original hypothesis [33]. Electron microscopy of NRV2 mice revealed significant RPE disturbances in the lesion of these animals (Fig. 7D). The changes of RPE morphology would be consistent with decreased oxygen supply to the outer retina due to reduced diffusion from the choroidal capillaries, subsequently leading to a rise in the ischemic factors, inflammatory factors, and retinal angiogenesis in this mouse strain. However, we did not observe any features of choroidal involvement, as seen in RCA, at any of the time points examined in NRV2 mice. Further observation is needed to elucidate the progression of RCA in this mouse model.

In conclusion, the origin and progression of angiogenesis observed in the NRV2 mouse strain closely resembles human RAP. The underlying etiopathogenesis, including genetic etiology and mode of disease progression in patients with RAP, is not well defined, in spite of several mouse models of RCA. Therefore, future studies using the NRV2 mouse could include genetic analysis that defines the specific genes and pathways involved in the angiogenic process, along with the role of potential growth factors that guide the neovessels to extend from the inner retina towards the choroid. This study begins to define a novel mouse model of ocular neovascularization that could become an important tool in developing future anti-angiogenic therapies.

## Supporting Information

Figure S1 **The fundus morphological changes found in NRV2 mice.** Fundus photographs and fluorescein angiography of NRV2 mice at p17 (*A*–*D)*, 2 M (*E, G)* and 3 M (*F, H)*. (*A, B*) Fundus images showed the emergence of depigmented regions at p17. (*C, D*) Fluorescein angiography did not show any vascular leakage corresponding to the areas of depigmentation at p17. (*E, F*) The depigmentation areas become faint at 2 M (*E*) and faded away at 3 M (*F*). (*G, H)* Fluorescein leakage almost disappeared after 2 M. Fluorescein angiography images were taken 3 minutes after fluorescein intraperitoneal injection; n = 5–10, Representative images are shown. p = postnatal day.
(TIF)

Figure S2 **H&E section of normal C57BL/6 mouse retina.** Retinal cross-sections of normal C57BL/6 mouse at p30 stained by H&E. (*A*) Cross-section from a C57BL/6 mouse showing the normal architecture of the retinal layers. (*B*) Higher magnification of (*A*) focusing on the normal architecture between the ONL and RPE interface. n = 3. Scale bars: 25 µm.
(TIF)

Figure S3 **VEGF and sFLT-1 protein expression in the retina of NRV2 mice.** (A) VEGF and (B) sFLT-1 protein concentration in the retina of NRV2 mice were quantified by ELISA and compared to age-matched C57BL/6 mice at several time points. (A) VEGF expression in NRV2 mice was significantly higher than C57BL/6 mice at p12, p17, p21, and p30. (B) sFLT-1 didn’t show any significant differences between NRV2 and C57BL/6. n = 3/timepoint *P<0.05, **P<0.01.
(TIF)

Movie S1 **The rotation image of 3D retinal vascular structure of NRV2 mouse at p15 from** **Figure 6C** **.**
(MOV)

Movie S2 **Confocal images were taken every 1 micron in the p25 retinal flat mount stained with isolectin B4.** Images were compiled sequentially, from superficial layers to deep layers, into video format. These images were used for making the 3D images of Fig. 6F and Movie S3.
(MOV)

Movie S3 **The rotation image of 3D retinal vascular structure of NRV2 mouse at p25 from** **Figure 6F** **.**
(MOV)
