# Supplementary figures and images for: Characterization of a Spontaneous Retinal Neovascular Mouse Model
Source: PLoS One. 2014 Sep 4;9(9):e106507. doi: 10.1371/journal.pone.0106507 (PMC4154693; doi:10.1371/journal.pone.0106507)

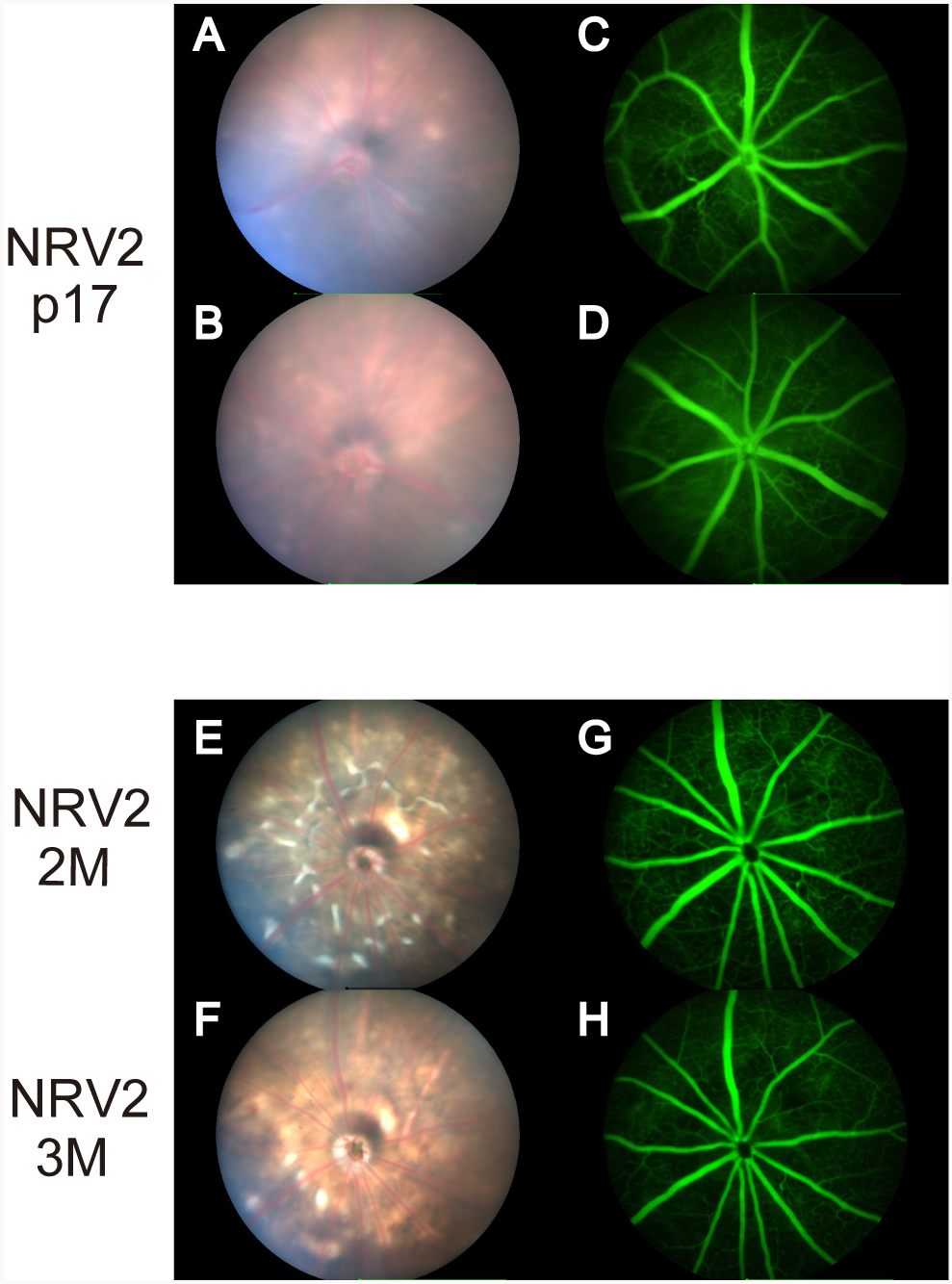

Supplement: Figure S1 — The fundus morphological changes found in NRV2 mice. Fundus photographs and fluorescein angiography of NRV2 mice at p17 (A–D), 2 M (E, G) and 3 M (F, H). (A, B) Fundus images showed the emergence of depigmented regions at p17. (C, D) Fluorescein angiography did not show any vascular leakage corresponding to the areas of depigmentation at p17. (E, F) The depigmentation areas become faint at 2 M (E) and faded away at 3 M (F). (G, H) Fluorescein leakage almost disappeared after 2 M. Fluorescein angiography images were taken 3 minutes after fluorescein intraperitoneal injection; n = 5–10, Representative images are shown. p = postnatal day. (TIF) [file pone.0106507.s001.tif]

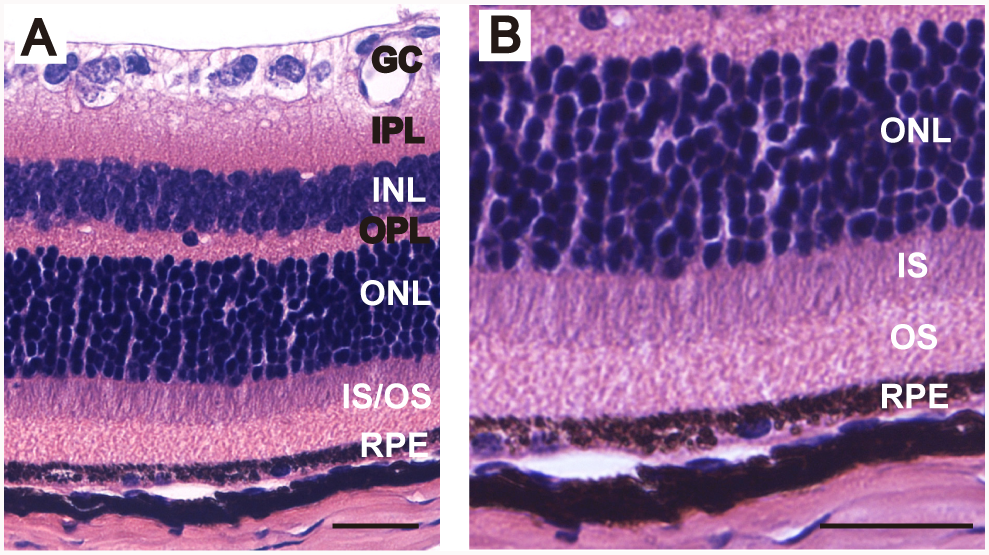

Supplement: Figure S2 — H&E section of normal C57BL/6 mouse retina. Retinal cross-sections of normal C57BL/6 mouse at p30 stained by H&E. (A) Cross-section from a C57BL/6 mouse showing the normal architecture of the retinal layers. (B) Higher magnification of (A) focusing on the normal architecture between the ONL and RPE interface. n = 3. Scale bars: 25 µm. (TIF) [file pone.0106507.s002.tif]

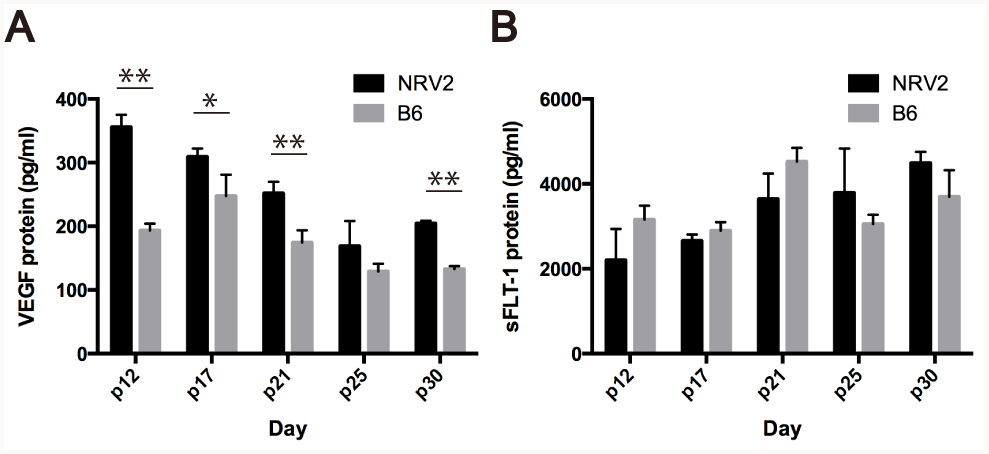

Supplement: Figure S3 — VEGF and sFLT-1 protein expression in the retina of NRV2 mice. (A) VEGF and (B) sFLT-1 protein concentration in the retina of NRV2 mice were quantified by ELISA and compared to age-matched C57BL/6 mice at several time points. (A) VEGF expression in NRV2 mice was significantly higher than C57BL/6 mice at p12, p17, p21, and p30. (B) sFLT-1 didn’t show any significant differences between NRV2 and C57BL/6. n = 3/timepoint *P<0.05, **P<0.01. (TIF) [file pone.0106507.s003.tif]
